# Detectability of intracranial vessel wall atherosclerosis using black-blood spectral CT: a phantom and clinical study

**DOI:** 10.1186/s41747-024-00473-x

**Published:** 2024-07-03

**Authors:** Fan Zhang, Hui Yao, Eran Langzam, Qinglin Meng, Xiao Meng, Rob J. van der Geest, Chuncai Luo, Tengyuan Zhang, Jianyong Li, Jianmei Xiong, Weiwei Deng, Ke Chen, Yangrui Zheng, Jingping Wu, Fang Cui, Li Yang

**Affiliations:** 1 Department of Radiology, Hainan Hospital of Chinese PLA General Hospital, Jianglin Road, Haitang District, Sanya, Hainan Province 572013 China; 2 The Second School of Clinical Medicine, Southern Medical University, Guangzhou, Guangdong Province China; 3 Philips CT Clinical Science, Philips Healthcare Global, Beijing, China; 4 Philips Healthcare Global, Haifa, Israel; 5 School of Health Industry Management, University of Sanya, Sanya, Hainan Province China; 6 Department of Radiology, Leiden University Medical Center, Leiden, The Netherlands; 7 Department of Radiology, The First Medical Center of Chinese PLA General Hospital, Beijing, China; 8 Department of Neurology, Hainan Hospital of Chinese PLA General Hospital, Sanya, Hainan Province China; 9 Philips Healthcare China, Shanghai, China; 10 Department of Neurosurgery, Hainan Hospital of Chinese PLA General Hospital, Sanya, Hainan Province China; 11 Department of Radiology, The Second Medical Center of Chinese PLA General Hospital, Beijing, China

**Keywords:** Arteries, Atherosclerosis, Computed tomography angiography, Phantoms (imaging), Stroke

## Abstract

**Background:**

Computed tomography (CT) is the usual modality for diagnosing stroke, but conventional CT angiography reconstructions have limitations.

**Methods:**

A phantom with tubes of known diameters and wall thickness was scanned for wall detectability, wall thickness, and contrast-to-noise ratio (CNR) on conventional and spectral black-blood (SBB) images. The clinical study included 34 stroke patients. Diagnostic certainty and conspicuity of normal/abnormal intracranial vessels using SBB were compared to conventional. Sensitivity/specificity/accuracy of SBB and conventional were compared for plaque detectability. CNR of the wall/lumen and quantitative comparison of remodeling index, plaque burden, and eccentricity were obtained for SBB imaging and high-resolution magnetic resonance imaging (hrMRI).

**Results:**

The phantom study showed improved detectability of tube walls using SBB (108/108, 100% *versus* conventional 81/108, 75%, *p* < 0.001). CNRs were 75.9 ± 62.6 (mean ± standard deviation) for wall/lumen and 22.0 ± 17.1 for wall/water using SBB and 26.4 ± 15.3 and 101.6 ± 62.5 using conventional. Clinical study demonstrated (i) improved certainty and conspicuity of the vessels using SBB *versus* conventional (certainty, median score 3 *versus* 0; conspicuity, median score 3 *versus* 1 (*p* < 0.001)), (ii) improved sensitivity/specificity/accuracy of plaque (≥ 1.0 mm) detectability (0.944/0.981/0.962 *versus* 0.239/0.743/0.495) (*p* < 0.001), (iii) higher wall/lumen CNR of SBB of (78.3 ± 50.4/79.3 ± 96.7) *versus* hrMRI (18.9 ± 8.4/24.1 ± 14.1) (*p* < 0.001), and (iv) excellent reproducibility of remodeling index, plaque burden, and eccentricity using SBB *versus* hrMRI (intraclass correlation coefficient 0.85–0.94).

**Conclusions:**

SBB can enhance the detectability of intracranial plaques with an accuracy similar to that of hrMRI.

**Relevance statement:**

This new spectral black-blood technique for the detection and characterization of intracranial vessel atherosclerotic disease could be a time-saving and cost-effective diagnostic step for clinical stroke patients. It may also facilitate prevention strategies for atherosclerosis.

**Key points:**

• Blooming artifacts can blur vessel wall morphology on conventional CT angiography.

• Spectral black-blood (SBB) images are generated from material decomposition from spectral CT.

• SBB images reduce blooming artifacts and noise and accurately detect small plaques.

**Graphical Abstract:**

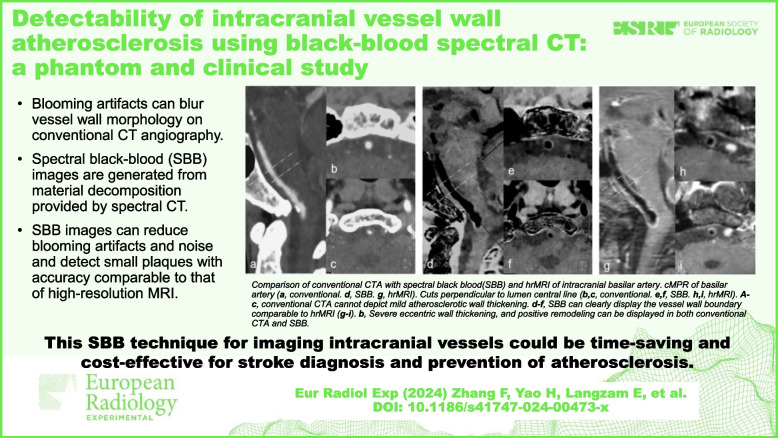

**Supplementary Information:**

The online version contains supplementary material available at 10.1186/s41747-024-00473-x.

## Background

Intracranial atherosclerotic disease is a leading etiology of ischemic stroke [1]. High-risk plaque with no or mild stenosis is more prevalent than previously acknowledged and is associated with embolic stroke of an undetermined source resulting in unfavorable outcomes [2, 3]. The diagnostic emphasis and assessment of intracranial atherosclerotic disease rather than intracranial stenosis are fundamental for risk stratification and rational decision-making with respect to available therapies. The common non-invasive technique for the detection of the disease is high-resolution magnetic resonance imaging (hrMRI) which serves as a “benchmark” for its detection. Yet, hrMRI requires long scan times and hence is prone to motion artifacts. In addition, hrMRI requires specialized receiver coils making it less applicable compared with computed tomography (CT) angiography (CT in clinical settings). Conventional CTA can provide arterial lumen and wall assessment and display plaque type, plaque density, and ulceration on relatively larger vessels such as the extracranial carotid and coronary arteries [4–9]. CT, however, is prone to blooming artifacts caused by high-attenuation iodinated contrast which may conceal the intima side of the vessel wall [10].

Dual-energy CT is a “spectral” technology that enables the acquisition of the anatomy at two different energy levels, which facilitate material decomposition. Prior work has shown that it can suppress the iodine inside the vascular lumen into “black” and can facilitate the depiction of the vessel wall. We call it “spectral black-blood” (SBB) reconstructions in this study. Rotzinger et al. [11] demonstrated the utility of this approach in enhancing the detectability of aortic intramural hematoma in the aortic wall. Si et al. [12] assessed the vascular involvement in pancreatic ductal adenocarcinoma using a similar approach.

Our study aimed to evaluate the feasibility and performance of both SBB and conventional reconstructions for the detection of intracranial plaque morphology compared to hrMRI as a reference standard.

## Methods

### Generation of the SBB results

The SBB results are generated by a material decomposition algorithm (IntelliSpace Portal version 12, Philips Healthcare, Best, The Netherlands). As seen in Additional file 1: Fig. S1, the spectral data provides, by definition, a representation of each of the voxels in two independent bases/axis (*x*–*y*), in our case an approximated photoelectric and Compton scatter components. The representation of each voxel in that plane is set in such a way that voxels with similar physical characteristics are clustered (*i.e.*, “scatter plots”).

In this algorithm, each voxel (*P* (*x*, *y*)) is projected in a constant direction to the air–water axis (*P* (*x*′, 0)). The direction of the projection was set based on preliminary tunings that look for a constant value that consistently brings iodine close to “black” (hence “air”), in our case, approximately 48°. This mathematical operation results in voxels representing high concentrations of iodine being suppressed and nearly zeroed (*i.e.*, “black”), while the voxels containing soft tissues remain unaffected. The volume that is created from this operation was referred to as “SBB” and served as the spectral result used for the plaque analysis.

### Phantom study

#### CT phantom

**Fig. 1:**
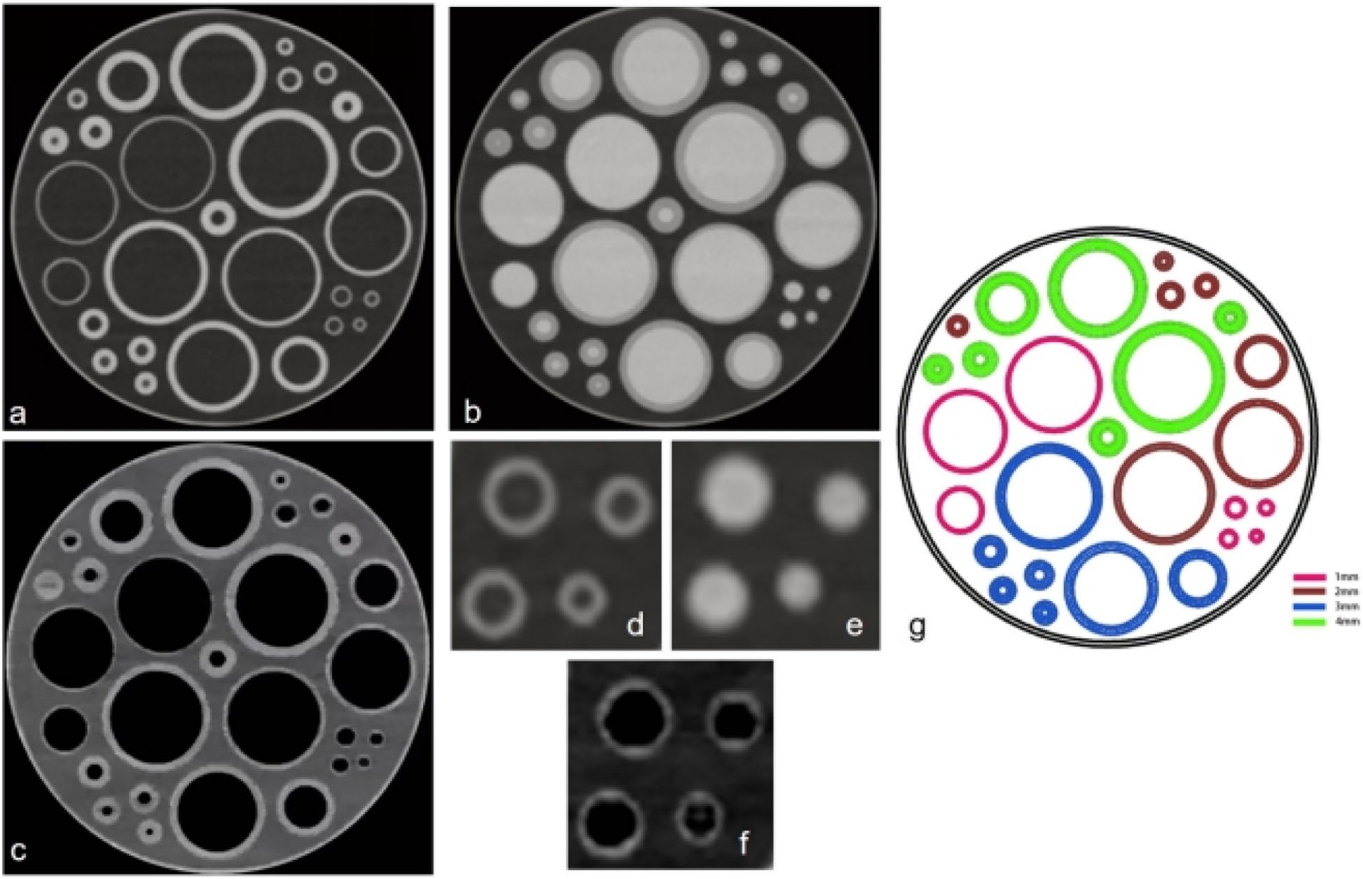
Phantom images. **a** Conventional image where all tubes and tank are filled with distilled water showing very well the tube wall boundaries. **b** Conventional image where all tubes are filled with iodinated solution and the tank is filled with distilled water. **c** SBB image where all tubes are filled with iodinated solution and the tank is filled with distilled water clearly showing the tube wall boundaries. **g** Ideograph of the phantom with different tube diameters. Partial views focused on the 1 mm wall thickness tubes: (**d**) conventional image where the tube is filled with water, (**e**) conventional image where the tube is filled with iodine (the tube wall boundary is not depicted clearly, especially in tube wall less than 1-mm diameter), and (**f**) SBB image where the tube is filled with iodinated solution. *SBB* Spectral black-blood

A three-dimensional (3D) custom-designed phantom was printed with light model resin with precise and durable characteristics (viscosity, 355 CPS at 28 °C; density, 1.13 g/cm^3^ at 25 °C; color, white; critical exposure, 9.3 MJ/cm^2^; curing depth, 0.145 mm; construction layer thickness, 0.1 mm; CT attenuation value, 252 HU). The phantom comprised 28 hollow tubes designed with inner diameters (“lumens”) of 3, 4, 5, 6, 16, 30, and 35 mm and wall thicknesses of 1, 2, 3, and 4 mm (Fig. 1). They were fixed in a 32-cm (diameter) cylindrical water tank. Since inner diameters of 3, 4, and 5 mm are within the range of intracranial basilar and vertebral artery lumen diameters, the analysis was limited to those inserts only. The tubes were filled with 5% iodine/95% distilled water-mixed contrast agent solution to create a mean CT attenuation value of 395 ± 32 HU (mean ± standard deviation) at 120 kVp. This value is consistent with typical CT values we see in the arteries in clinical CTA scans.

#### Data acquisition

The phantom was scanned using the cervicocerebral CTA protocols that are typically used in patient exams. Scans were performed on a dual-layer spectral detector CT scanner (IQon CT; Philips Healthcare, Best, The Netherlands) using the following acquisition parameters to cover a typical range of patient scans:

i. Collimation 64 × 0.625 mm
ii. Gantry rotation time 0.27 s
iii. Helical pitch 0.9
iv. Tube voltage 120 kVp
v. Tube current 50, 80, and 200 mAs (representing low, mid, and high levels of radiation dose)

Each scan was repeated three times to evaluate the interscan precision; overall, nine datasets were acquired.

#### Post-processing

Both conventional and SBB images (reconstructed at 0.67 mm) were generated from the spectral-based image. The conventional images were reconstructed using the hybrid interactive reconstruction algorithm “iDose^4^” while SBB images were reconstructed using a dedicated spectral reconstruction algorithm.

#### Measurements

The derived datasets were used to perform qualitative and quantitative assessments.

The qualitative assessment focused on whether the tube wall was detected or not. Quantitatively, the accuracy (mean absolute error [MAE] and error percentage) of tube wall size measurement was assessed by measuring the tube’s inner diameter and wall thickness and comparing them to the known tube specifications. The following were measured:

- 1. Accuracy of wall thickness (conventional and SBB)
- 2. Accuracy of inner diameter (conventional and SBB)
- 3a. Contrast-to-noise ratio (CNR) of tube lumen against tube wall (conventional and SBB)
- 3b. CNR of tube wall against container background (water)

A dedicated software (VesselMass version 5.0, Leiden University Medical Center, Leiden, The Netherlands) was used to extract the measurements of the average tube wall thickness and diameter automatically. The window level and width were set to 220 HU and 60 HU for conventional and 580 HU and 360 HU for SBB, respectively. Two concentric regions of interest (ROIs) encompassing the lumen and tube wall were outlined by a radiologist with 15 years of experience in cardiovascular imaging (FZ) to extract pixel values of the tube lumen and wall; additionally, one ROI was placed in the container background. The software reports the quantitative results automatically. The observer calculated the CNR using the following formulas:

$$\begin{array}{c}\left(3{\text{a}}\right){{\text{CNR}}}_{\mathrm{lumen\ against\ wall}}=\frac{\left|\left(\mathrm{mean\ }{{\text{ROI}}}_{{\text{lumen}}}-\mathrm{mean\ }{{\text{ROI}}}_{{\text{wall}}}\right)\right|}{\sqrt{\frac{1}{2}\left({{{\text{SD}}}^{2}}_{{\text{lumen}}}+{{{\text{SD}}}^{2}}_{{\text{wall}}}\right)}}\\ \left(3{\text{b}}\right){{\text{CNR}}}_{\mathrm{wall\ against\ water}}=\frac{\left|\left(\mathrm{mean\ }{{\text{ROI}}}_{{\text{wall}}}-\mathrm{mean\ }{{\text{ROI}}}_{{\text{water}}}\right)\right|}{\sqrt{\frac{1}{2}\left({{{\text{SD}}}^{2}}_{{\text{wall}}}+{{{\text{SD}}}^{2}}_{{\text{water}}}\right)}}\end{array}$$

### Clinical study

#### Study population

This study was approved by our institutional review board ethics committee. Informed consent was waived for spectral CTA for this retrospective study. The inclusion criteria were age ≥ 18 years, patients with acute ischemic stroke within 24 h from symptoms onset, patients with stroke history clinically indicated for performing CTA due to recent symptoms, patients that came in for a physical examination and CTA was performed due to transient ischemic attack, and patients who agreed to have hrMRI within one week after the CT scan. The exclusion criteria were patients with a metal implant in the head and/or neck, patients with malignant tumors in any part of the body, patients who underwent previous head/neck radiation therapy, patients with balloon dilatation and stent implantation, patients with total occlusion in one of their cranio-vascular arteries segments, and calcified or mixed plaques.

Out of 1,095 consecutive patients admitted to our hospital between December 2018 and March 2021, 103 patients had both CTA and hrMRI scans. Out of those, 45 patients were excluded due to the presence of metal artifacts around the skull or neck area (3 cases) or motion artifacts (42 cases), and 24 patients were excluded due to the presence of total occlusion. The remaining 34 patients were enrolled in our study.

#### Image acquisition and post-processing

All cervicocerebral CTA examinations were performed in a supine position from the aortic arch up to the skull vertex. The acquisition parameters were identical to those used in the phantom study but with a tube current of 125–150 mAs (per dose modulation recommendation); 50–60 mL of contrast agent (Ultravist Solution 370 mg I/mL; Bayer Healthcare, Berlin, Germany) was intravenously injected through an antecubital vein with an injection flow rate of 3.5–4 mL/s followed by 50 mL of saline solution at the same flow rate. Injection timing was controlled by the bolus-tracking technique with tracker ROI placed in the ascending aorta (threshold set to 120 HU). The volume CT dose index was 33.9 ± 12.1 mGy (mean ± standard deviation); the dose length product was 1,504.5 ± 513.7 mGy × cm; the effective dose was 4.7 ± 1.6 mSv [13].

Cervicocerebral hrMRI scans were performed on a 3-T scanner (Philips Ingenia CX, Best, The Netherlands) with a custom-designed 40-channel neurovascular coil. Unenhanced T1-weighted VISTA, T2-weighted VISTA, and contrast-enhanced T1-weighted VISTA were utilized as a set of 3D multicontrast protocols providing T1 and T2 vessel wall images. All sequences were performed at identical scan planes and with the same spatial resolution covering the cervicocerebral artery. All imaging parameters for both reference and 3D protocols have been previously described [14]. The 3D multicontrast protocol can achieve isotropic 0.8 mm × 0.8 mm × 0.8 mm = 0.512 mm^3^ resolution to cover the major intracranial and entire carotid arteries within 30–40 min.

Conventional CTA and SBB images were reconstructed with parameters similar to those used in the phantom study. Conventional CTA and SBB images of the proximal, mid, and distal basilar arteries and bilateral vertebral artery were reviewed side by side, with hrMRI images of the same vessels reviewed separately using the VesselMass software. For conventional CTA, SBB, and hrMRI, curved planar reformatted images were generated to evaluate the cervicocerebral arteries. The images were coregistered, and then, a perpendicular section was selected. Cross-sectional cuts perpendicular to the lumen central line were used to evaluate the vessel wall. Cross-sectional views of the vessel wall on conventional CTA, SBB images, and hrMRI images were reconstructed following the post-processing described above.

#### Image reading

All images were read independently by two experienced radiologists (H.Y. and F.Z., with 10 and 15 years of experience in cardiovascular imaging, respectively) and two relatively inexperienced radiologists (M.X. and J.W., each with 2 years of experience in cardiovascular imaging). The wall thickness was rated according to hrMRI as > 1.0 mm, > 1.5 mm, or > 2 mm for both conventional CTA and SBB. The experienced readers reviewed the images once, and the two inexperienced readers reviewed the datasets twice: the first time without any dedicated training and the second time > 4 weeks after being trained by experienced users. All observers were blinded to the clinical information. Study images were reviewed in random order. The window level and width were adjusted by the reader per personal preferences. Qualitative image quality assessment of diagnostic certainty and conspicuity of plaque in conventional and SBB datasets were performed by the two experienced readers.

Diagnostic certainty was defined using a 5-point Likert scale where 0 was the unclear vessel wall boundary and uncertain diagnosis, 1 was the ambiguous vessel wall boundary or atherosclerosis and low probability of diagnosis, 2 was the intermediate level of wall boundary display or atherosclerosis and moderate probability of diagnosis, 3 was the apparent vessel wall or atherosclerosis and high probability of diagnosis, and 4 was the clear vessel wall or atherosclerosis, and definite diagnosis could be determined. Conspicuity of the inner and outer wall was defined using a 4-point Likert scale where 0 represented the obscured margin, 1 represented the clear inner wall but unclear outer wall, 2 where most of both inner and outer walls were clear, and 3 represented the perfect circumferential wall display of both inner and outer wall.

Quantitative plaque detectability on conventional and SBB images was assessed by both experienced and inexperienced readers. In these tests, the reviewers were asked to detect plaques in the basilar, right vertebral artery V4 segment, and left vertebral artery V4 segment, on conventional, SBB, and hrMRI independently. The results derived from the CT images were then compared to the hrMRI benchmark for true positive, true negative, false positive, false negative, sensitivity, specificity, and accuracy. To keep a segment-based weighting and for a segment where there was more than one plaque, we weighted each based on the overall number of plaques in the segment (*e.g.*, if there were three plaques present, each accounted for 1/3). This was done in order to not overweight segments with multiple plaques over those that only have a single plaque.

ROIs in the clinical study were placed the same way as in the phantom study. ROI of the background was placed in the periarterial cerebrospinal fluid (CSF). CNR of both normal and abnormal vessels were calculated using the following formulas:

$$\begin{array}{c}{{\text{CNR}}}_{\mathrm{lumen\ against\ wall}}=\frac{\mathrm{mean\ }{{\text{ROI}}}_{{\text{lumen}}}-\mathrm{mean\ }{{\text{ROI}}}_{{\text{wall}}}}{\sqrt{\frac{1}{2}\left({{{\text{SD}}}^{2}}_{{\text{lumen}}}+{{{\text{SD}}}^{2}}_{{\text{wall}}}\right)}}\\ {{\text{CNR}}}_{\mathrm{wall\ against\ CSF}}=\frac{\mathrm{mean\ }{{\text{ROI}}}_{{\text{wall}}}-\mathrm{mean\ }{{\text{ROI}}}_{{\text{CSF}}}}{\sqrt{\frac{1}{2}\left({{{\text{SD}}}^{2}}_{{\text{wall}}}+{{{\text{SD}}}^{2}}_{{\text{CSF}}}\right)}}\end{array}$$

Quantitative measures of morphology features such as remodeling index, plaque burden, and eccentricity were extracted from the images and compared to hrMRI. Each image was magnified four to six times with bilinear interpolation. The lumen and outer wall boundaries were traced manually along the interfaces between the lumen and wall and between the wall and surrounding tissue respectively. When part of a boundary was invisible, the contour was completed to maintain the continuity of the vessel’s curvature. The software extracted measurements of the average and maximum wall thickness (*i.e.*, the mean and maximum value of the distances between the lumen and wall contours), the lumen area (*i.e.*, the area inside the luminal contour), and the wall area (*i.e*., subtracting the inner contour area from the outer contour area). The experienced radiologist manually selected three cross-sections along the vessel: narrowest, healthy proximal, and healthy distal where measurements were performed, and the lumen area and outer wall area were manually extracted. The wall area was calculated as outer wall area minus lumen area for each of the identified cross-sections. Morphological features were obtained as follows: the remodeling index was calculated as outer wall area lesion/outer wall area reference; plaque burden as (wall area/outer wall area at the site of maximal lumen narrowing) %, and eccentricity as (wall thickness maximal minus wall thickness minimal)/wall thickness maximal.

### Statistical analysis

Statistical analysis was performed using commercially available software SPSS version 25.0 (IBM Corp., NY USA) and Prism 9 (GraphPad Software, Inc., SD, USA). Continuous variables were expressed as mean, standard deviation (SD), MAE, SD error, and coefficient of variance (CV) while categorical variables were expressed as a value or percentage. Sensitivity, specificity, accuracy, positive predictive value, and negative predictive value were determined. Comparisons between the groups were performed using the one-way analysis of variance for continuous variables with normal distributions, and the Kruskal–Wallis test was used for continuous variables with non-normal distributions. The *χ*^2^ test was used to analyze the categorical variables. Diagnostic certainty, lesion conspicuity, and CNR boxplot were conducted by using Prism 9. Classifications of diagnostic certainty and lesion conspicuity to interpret the strength of the agreement were based on the Cohen *κ* value, with values ≤ 0 indicating no agreement, 0.01–0.20 as none to slight, 0.21–0.40 as fair, 0.41–0.60 as moderate, 0.61–0.80 as substantial, and 0.81–1.00 as almost perfect agreement. Likert scores are listed as medians and interquartile ranges (IQRs). Intraclass correlation coefficient (ICC) provided the level of reliability using the following general guidelines: values less than 0.50 are indicative of poor reliability, values between 0.50 and 0.75 indicate moderate reliability, values between 0.75 and 0.90 indicate good reliability, and values greater than 0.90 indicate excellent reliability.

## Results

### Phantom study

#### Wall detectability

Out of the 108 samples that were acquired (3 dose levels × 12 inserts × 3 independent measurements per insert), the detectability of tube wall was 100% (108/108) in the SBB images and 75% (81/108) using conventional CTA images (*p* < 0.001). The tube wall thickness of 1.0 mm was not detected at all in the conventional CTA images (0%, 0/27) (Fig. 1e), while they were fully detected on the SBB images (100%, 27/27) (Fig. 1f). For all other wall thicknesses, the performance of both conventional and SBB was the same (100%, 81/81).

#### Wall thickness and tube inner diameter

Overall, 108 samples were acquired for either tube inner diameter or wall thickness, including 81 samples for the conventional images and 108 samples for the SBB images (Tables 1 and 2). The MAE values for the measurement of the tube inner diameter were -0.73 ± 0.27 mm (error relative to tube specification [*i.e.*, the nominal value declared by the manufacturer] 19.0%) for the conventional images and -0.03 ± 0.11 mm (error relative to specification 2.0%) (*p* < 0.001) for the SBB images. The MAE values for measurement of the tube wall thickness were 0.18 ± 0.23 mm (error relative to tube specification 8.0%) for the conventional images and -0.03 ± 0.11 mm for the SBB images (error relative to tube specification (accuracy) 3.0%) (*p* < 0.001) (Tables 3 and 4). As a complementary analysis, CV was calculated for the sampled inner tube diameter and tube wall thickness dimensions. The derived CV values for a tube inner diameter of 3, 4, and 5 mm, were 0.08, 0.07, and 0.08 for the conventional and 0.03, 0.03, and 0.02 for the SBB, respectively. The derived CV values for the tube wall thickness of 1, 2, 3, and 4 mm were not assessed, 0.07, 0.06, and 0.08 for the conventional and 0.08, 0.05, 0.03, and 0.02 for the SBB, respectively (Tables 1 and 2).

**Table 1:** Phantom tube inner diameter measurements (“tube lumen”)

	Tube inner diameter (mm)	Number	Mean	Standard deviation	Coefficient of variation
Conventional	3.00	27	2.20	0.17	0.08
	4.00	27	3.30	0.24	0.07
	5.00	27	4.31	0.34	0.08
SBB	3.00	36	3.04	0.10	0.03
	4.00	36	4.03	0.12	0.03
	5.00	36	5.01	0.11	0.02

*SBB* Spectral black-blood

**Table 2:** Phantom tube wall thickness diameter measurements (“tube wall”)

	Tube wall thickness (mm)	Number	Mean	Standard deviation	Coefficient of variation
Conventional	1.00	0	–	–	–
	2.00	27	2.17	0.15	0.07
	3.00	27	3.27	0.20	0.06
	4.00	27	4.09	0.30	0.06
SBB	1.00	27	1.01	0.08	0.08
	2.00	27	2.08	0.10	0.05
	3.00	27	3.10	0.09	0.03
	4.00	27	3.98	0.06	0.02

*SBB* Spectral black-blood

**Table 3:** Tube inner diameter measurements: mean absolute error (MAE) and standard deviation (SD) errors expressed in millimeters and percentage

Conventional	MAE (mm)	-0.73	MAE (%)	19.0%
	SD error (mm)	0.27	SD error (%)	8.2%
SBB	MAE (mm)	0.04	MAE (%)	2.0%
	SD error (mm)	0.09	SD error (%)	2.1%

*SBB* Spectral black-blood

**Table 4:** Tube wall thickness measurements: mean absolute error (MAE) and standard deviation (SD) errors expressed in millimeters and percentage

Conventional	MAE (mm)	0.18	MAE (%)	8.0%
	SD error (mm)	0.23	SD error (%)	5.7%
SBB	MAE (mm)	0.03	MAE (%)	3.0%
	SD error (mm)	0.11	SD error (%)	4.4%

*SBB* Spectral black-blood

#### CNR measurements

**Fig. 2:**
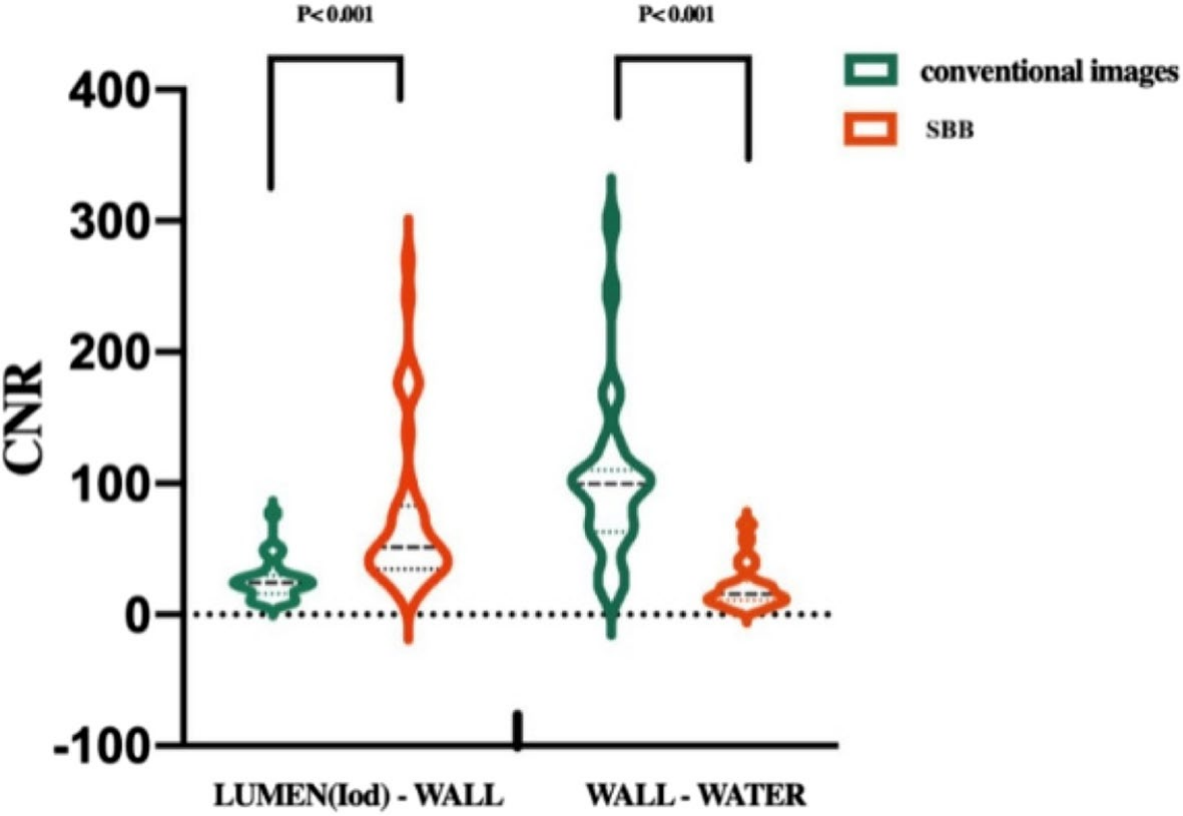
Violin plot shows phantom CNR of the lumen to wall and wall to water of conventional compared to SBB images. CNR of the lumen to wall in SBB is significantly higher than that in conventional images due to the high-density iodine suppression by the SBB technique. *CNR* Contrast-to-noise ratio, *SBB* Spectral black-blood

The derived values of CNR for the SBB were 75.98 ± 62.62 and 21.95 ± 17.13 for the wall/lumen and wall/water, respectively. In comparison, the derived values for the conventional images were 26.42 ± 15.30 and 101.6 ± 62.49 for the wall/lumen and wall/water, respectively (Table 5). The SBB provided a significantly higher CNR value of wall to lumen than the conventional images (Fig. 2).

**Table 5:** Phantom measurements of the CNR between the lumen and wall, and wall and water surroundings

	Contrast-to-noise ratio			
	Lumen (iodine filled)-to-wall		Wall-to-water	
	Mean	Standard deviation	Mean	Standard deviation
Conventional	26.4	15.3	101.6	62.5
SBB	76.0	62.6	22.0	17.1

### Clinical study

#### Patient characteristics

Overall, 34 patients (22 men, 12 women) were included in this study. Table 6 summarizes the key characteristics of the patient demographics.

**Table 6:** Overview of patient demographics and clinical data

Characteristic		*n* = 34	Range (min–max)
Age (years), mean ± standard deviation		64 ± 10	38–81
Women/(women + men), *n*/*n* (%)		12/34 (35)	–
Body mass index, mean ± SD		26.1 ± 3.1	–
Hypercholesterolemia, *n* (%)		12/34 (35)	–
Hypertension, *n* (%)		19/34 (56)	–
Diabetes mellitus, *n* (%)		13/34 (38)	–
Smoking, *n* (%)		10/34 (29)	–
Use of statins, *n* (%)		7/34 (21)	–
Three or more risk factors, *n* (%)		11/34 (32)	–
TIA/stroke, *n* (%)		25/34 (74)	–
TIA/stroke, *n* (%)	Anterior circulation, *n* (%)	–	–
	Posterior circulation, *n* (%)	8/25 (32)	–
Cholesterol (mmol/L)		4.51 ± 0.94	2.84–6.55
Triglycerides (mmol/L)		2.01 ± 1.31	0.5–7.0
HDL (mmol/L)		1.18 ± 0.25	0.68–1.82
LDL (mmol/L)		3.52 ± 0.68	1.48–24.49
ALT (U/L)		26.18 ± 12.45	9.5–55.9
AST (U/L)		21.75 ± 6.01	13.4–44.7
Bilirubin total (μmol/L)		11.06 ± 4.22	4.4–22.1
Glucose (mmol/L)		5.80 ± 1.15	4.13–10.69
Homocysteine (μmol/L)		13.88 ± 5.41	5.95–28.4
BUN (mmol/L)		5.25 ± 1.29	3.24–8.64
Creatinine (μmol/L)		69.37 ± 10.12	46.0–88.6
Uric acid (μmol/L)		381.44 ± 81.85	216–600
hsCRP/C-reactive (g/dL)		0.42 ± 0.21	0.02–0.85
Plasma fibrinogen (g/L)		3.22 ± 1.68	2.0–12.7
Troponin T (ng/mL)		0.005 ± 0.002	0.00–0.01
Brain natriuretic peptide (pg/mL)		21.26 ± 18.18	1.40–96.00
CK total (U/L)		165.93 ± 20.52	141.3–211
CK-MB mass (U/L)		16.03 ± 11.39	9.0–79.9

*ALT* Alanine aminotransferase, *AST* Aspartate aminotransferase, *BUN* Blood urea nitrogen, *CK-MB* Creatine kinase-myocardial band, *HDL* High-density lipoprotein, *hsCRP/C* High-sensitive C-reactive protein, *LDL* Low-density lipoprotein, *TIA* Transient ischemic attack

#### Qualitative analysis: diagnostic certainty and artery conspicuity

**Fig. 3:**
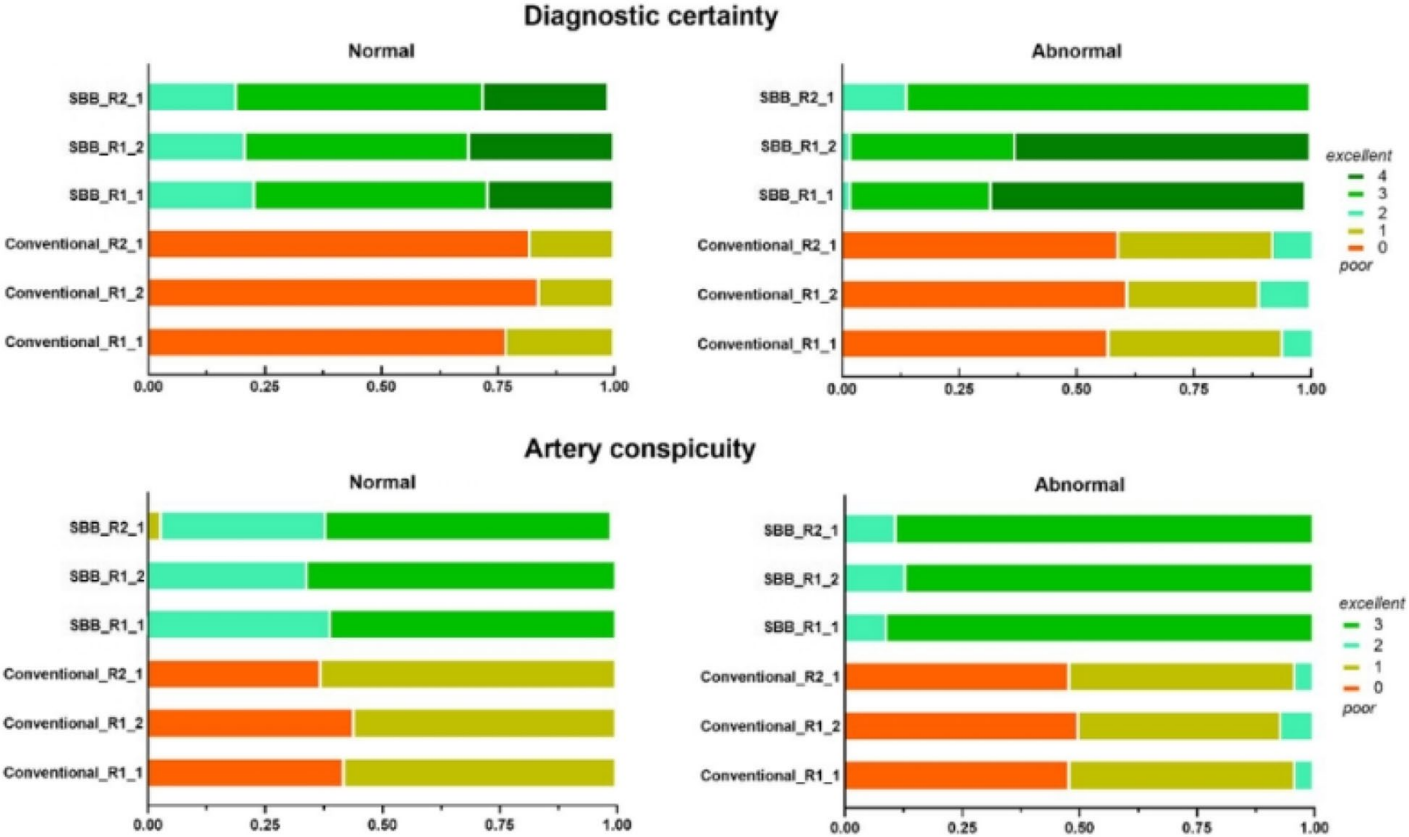
Subjective scores for the assessment of intracranial vessels for both conventional and SBB from two readers (two readings from reader 1). Higher scores were achieved from SBB for both diagnostic certainty and vessel conspicuity. *SBB* Spectral black-blood

Interobserver and intraobserver agreements on the diagnostic certainty and conspicuity of SBB were similar to conventional for healthy vessel segments and improved for abnormal vessel segments (see *κ* analysis, Table 7). Compared to conventional CTA images, SBB revealed a higher median diagnostic certainty in normal and diseased arteries (score 3, IQR 3–4; score 4, IQR 3–4 *versus* 0, IQR 0–1; and score 0, IQR 0–0, respectively; *p* < 0.001) as well as a higher artery conspicuity (score 3, IQR 2–3; score 3, IQR 3–3 *versus* score 1, IQR 0–1; and score 1, IQR 0–1, respectively; *p* < 0.001). The mean values between readers were obtained for quantitative measures; consensus was reached for qualitative features. The results of the subjective assessment are illustrated in Fig. 3.

**Table 7:** The inter-reader and intra-reader agreement of normal and abnormal arteries on diagnostic certainty and artery conspicuity from conventional and spectral black-blood (SBB) images

Inter-reader agreement				Intra-reader agreement			
		*κ*	95% CI			*κ*	95% CI
Diagnostic certainty							
Normal	SBB	0.78	0.65–0.91	Normal	SBB	0.89	0.81–0.98
	Conventional	0.76	0.54–0.98		Conventional	0.79	0.60–0.99
Abnormal	SBB	0.93	0.84–1.00	Abnormal	SBB	0.87	0.74–0.99
	Conventional	0.83	0.68–0.98		Conventional	0.83	0.66–0.99
Artery conspicuity							
Normal	SBB	0.76	0.60–0.93	Normal	SBB	0.90	0.79–1.00
	Conventional	0.71	0.54 to 0.87		Conventional	0.90	0.78–1.00
Abnormal	SBB	0.93	0.83 to 1.00	Abnormal	SBB	0.93	0.83–1.00
	Conventional	0.74	0.46–1.00		Conventional	0.84	0.62–1.00

*CI* Confidence interval

#### Quantitative analyses: plaque detectability

**Fig. 4:**
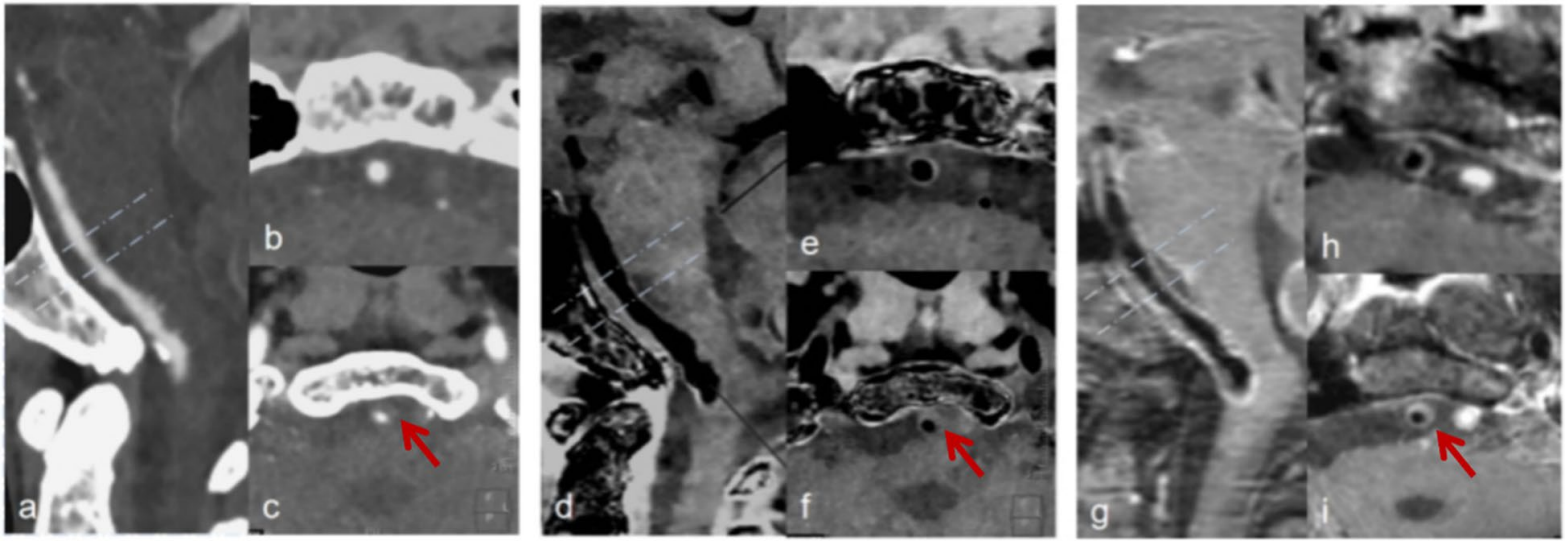
Comparison of conventional CTA with SBB and hrMRI of intracranial basilar artery. Male, 72 years old with transient consciousness disorder. Sagittal MPR of the basilar artery: (**a**) conventional, (**d**) SBB, and (**g**) hrMRI. Cross-sectional cuts perpendicular to lumen central line: (**b**, **c**) conventional, (**e**, **f**) SBB, and (**h**, **i**) hrMRI. While conventional CTA cannot depict mild atherosclerotic wall thickening, SBB can clearly display the vessel wall boundary comparable to hrMRI. Severe eccentric wall thickening and positive remodeling can be displayed in both conventional CTA and SBB, similar to hrMRI (arrows in **c**, **f**, and **i**), but SBB provided better diagnostic certainty and conspicuity. *CTA* Computed tomography angiography, *hrMRI* High-resolution magnetic resonance imaging, *SBB* Spectral black-blood

In our sample, the conventional CTA did not depict the mild atherosclerotic wall thickening in the intracranial artery (Fig. 4b). However, SBB (Fig. 4e) could clearly display the aforementioned vessel wall boundary comparable to hrMRI (Fig. 4h). For the vessel wall with a severe positive remodeling, the SBB showed a better depiction of the inner and outer boundaries (Fig. 4f) compared to conventional (Fig. 4c) and comparable to hrMRI (Fig. 4i). In the subjective assessment of the two experienced readers, sensitivity, specificity, and accuracy of plaque detectability (> 1.0 mm) using SBB were 94% (47.3/50.2), 98% (50.8/51.8), and 96% (98.1/102) *versus* 24% (12/50.2), 74% (38.3/51.8), and 50% (50.5/102), respectively, from conventional images (*p* < 0.001) (Tables 10 and 11). In the subjective assessment performed by the two inexperienced readers before training, sensitivity, specificity, and accuracy of plaque detectability (> 1.0 mm) using SBB were 85% (42.8/50.2), 81% (41.8/51.8), and 83% (84.6/102) *versus* 25% (12/50.1), 72% (37.5/51.8), and 49% (49.5/102), respectively, from conventional images (*p* < 0.001) (Tables 8 and 11). In contrast, following training, sensitivity, specificity, and accuracy of plaque detectability (> 1.0 mm) using SBB were 89% (44.8/50.2), 96% (49.8/51.8), and 93% (94.6/102) *versus* 22% (11/50.2), 70% (36.5/51.8), and 47% (47.5/102), respectively from conventional images (*p* < 0.001) (Tables 9, 10, and 11).

**Table 8:** Sensitivity, specificity, and accuracy for inexperienced readers, before training

Plaque size (mm)	Conventional or SBB	TP	TN	FP	FN	Overall positive	Overall negative	Overall samples	Sensitivity	Specificity	Accuracy	*p*-value
> 1.0	Conventional	**12.0**	**37.5**	**14.3**	**38.2**	**50.2**	**51.8**	102	0.239	0.723	0.485	< 0.0001
> 1.0	SBB	**42.8**	**41.8**	**10.0**	**7.3**	**50.2**	**51.8**	102	0.854	0.807	0.830	
> 1.5	Conventional	**11.5**	**37.5**	**14.5**	**25.5**	37	52	89	0.311	0.721	0.551	< 0.0001
> 1.5	SBB	**35.5**	**42**	**10**	**1.5**	37	52	89	0.959	0.808	0.871	
> 2.0	Conventional	**7.5**	**38**	**15**	**11.5**	19	53	72	0.395	0.717	0.632	< 0.001
> 2.0	SBB	**18.5**	**43**	**10**	**0.5**	19	53	72	0.974	0.811	0.854	

Values in bold are normalized to reflect the segment-level weighting (*e.g.*, if there were three plaques present, each accounted for 1/3). For details, please see “Quantitative plaque detectability” in the “Methods” section

*FP* False positives, *FN* False negatives, *SBB* Spectral black-blood, *TN* True negatives, *TP* True positives

**Table 9:** Sensitivity, specificity, and accuracy for inexperienced readers, after training

Plaque size (mm)	Conventional or SBB	TP	TN	FP	FN	Overall positive	Overall negative	Overall samples	Sensitivity	Specificity	Accuracy	*p*-value
> 1.0	Conventional	**11.0**	**36.5**	**15.3**	**39.2**	**50.2**	**51.8**	102	0.219	0.704	0.466	< 0.0001
> 1.0	SBB	**44.8**	**49.8**	**2.0**	**5.3**	**50.2**	**51.8**	102	0.894	0.961	0.928	
> 1.5	Conventional	**10**	**36.5**	**15.5**	**27**	37	52	89	0.270	0.702	0.522	< 0.0001
> 1.5	SBB	**34**	**50**	**2**	**3**	37	52	89	0.919	0.962	0.944	
> 2.0	Conventional	**6**	**37**	**16**	**13**	19	53	72	0.316	0.698	0.597	< 0.0001
> 2.0	SBB	**17.5**	**51**	**2**	**1.5**	19	53	72	0.921	0.962	0.951	

Values in bold are normalized to reflect the segment-level weighting (*e.g.*, if there were three plaques present, each accounted for 1/3). For details, please see “Quantitative plaque detectability” in the “Methods” section

*FP* False positives, *FN* False negatives, *SBB* Spectral black-blood, *TN* True negatives, *TP* True positives

**Table 10:** Sensitivity, specificity, and accuracy for experienced readers

Plaque size (mm)	Conventional or SBB	TP	TN	FP	FN	Overall positive	Overall negative	Overall samples	Sensitivity	Specificity	Accuracy	*p*-value
> 1.0	Conventional	**12.0**	**38.5**	**13.3**	**38.2**	**50.2**	**51.8**	102	0.239	0.743	0.495	< 0.0001
> 1.0	SBB	**47.3**	**50.8**	**1.0**	**2.8**	**50.2**	**51.8**	102	0.944	0.981	0.962	
> 1.5	Conventional	**11.5**	**38.5**	**13.5**	**25.5**	37	52	89	0.311	0.740	0.562	< 0.0001
> 1.5	SBB	**35.5**	**51**	**1**	**1.5**	37	52	89	0.959	0.981	0.972	
> 2.0	Conventional	**7.5**	**39**	**14**	**11.5**	19	53	72	0.395	0.736	0.646	< 0.0001
> 2.0	SBB	**18.5**	**52**	**1**	**0.5**	19	53	72	0.974	0.981	0.979	

Values in bold are normalized to reflect the segment-level weighting (*e.g.*, if there were three plaques present, each accounted for 1/3). For details, please see “Quantitative plaque detectability” in the “Methods” section

*FP* False positives, *FN* False negatives, *SBB* Spectral black-blood, *TN* True negatives, *TP* True positives

**Table 11:** Summary of accuracy for the different results and reviewer experience and state

Accuracy	Plaque size		
	> 1.0 mm	> 1.5 mm	> 2.0 mm
Inexperienced, before training, conventional	0.485	0.551	0.632
Inexperienced, before training SBB	0.830	0.871	0.854
Inexperienced, after training, conventional	0.466	0.522	0.597
Inexperienced, after training, SBB	0.928	0.944	0.951
Experienced, conventional	0.495	0.562	0.646
Experienced, SBB	0.962	0.972	0.979

*SBB* Spectral black-blood

#### Quantitative analysis: CNR of wall/lumen and wall/periarterial CSF

**Fig. 5:**
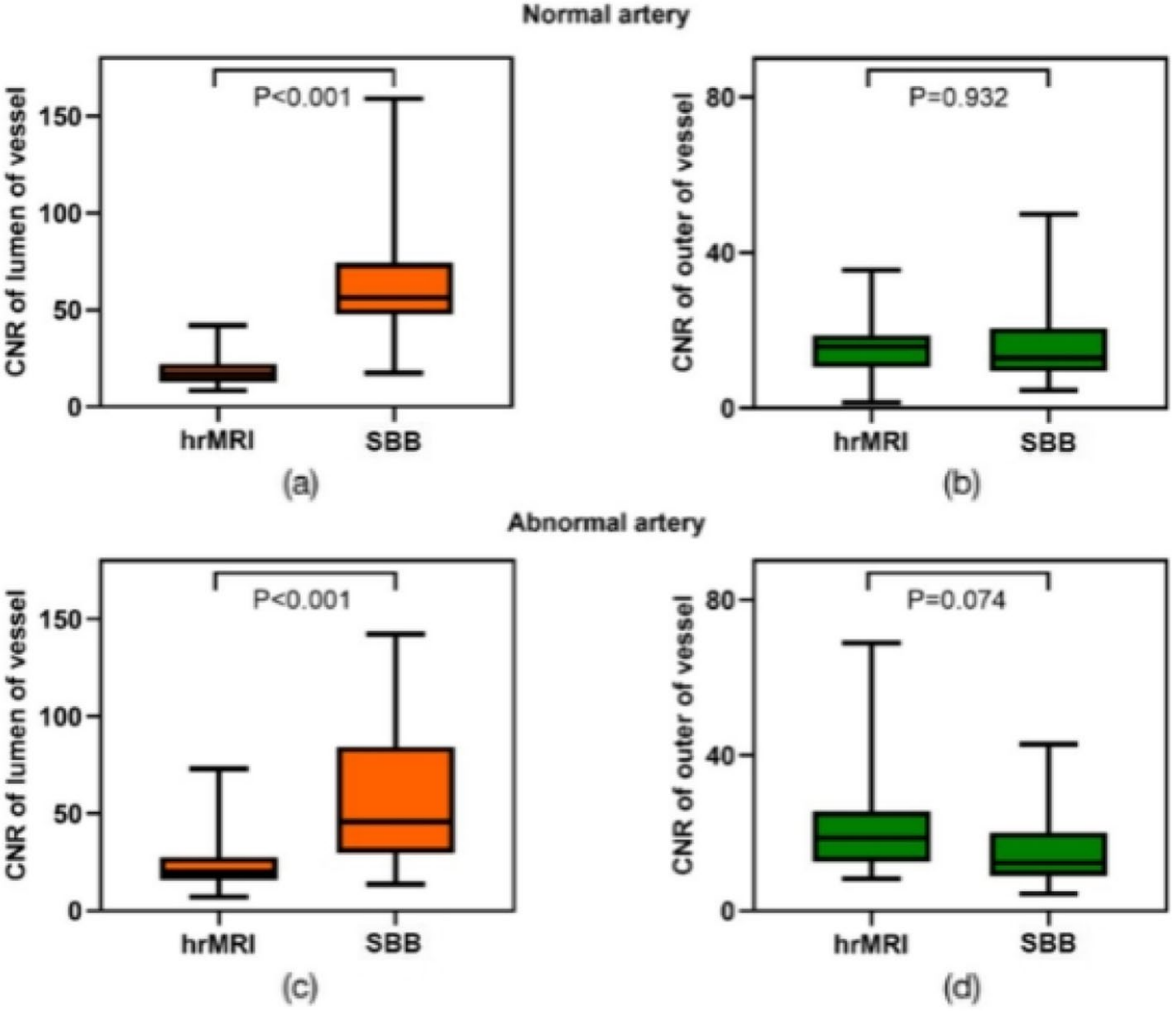
Box-whisker plots show CNR of the intracranial vessels from hrMRI and SBB. **a**, **b** CNR of the lumen and outer of normal vessels. **c**, **d** CNR of the lumen and outer of abnormal vessels. On both normal and abnormal vessels, CNR_wall/lumen_ of SBB are significantly higher than those of hrMRI. There is no significant difference in CNR_wall/periarterial CSF_ between SBB and hrMRI. *CNR* Contrast-to-noise ratio, *CTA* Computed tomography angiography, *hrMRI* High-resolution magnetic resonance imaging, *SBB* Spectral black-blood

CNR analysis of the SBB relative to hrMRI demonstrated that for both normal and abnormal vessels, CNR of wall/lumen of SBB (78.3 ± 50.4, 79.3 ± 96.7) was significantly higher than seen on hrMRI (18.9 ± 8.4, 24.1 ± 14.1) (*p* < 0.001 for both) while CNR of wall/periarterial CSF in SBB (16.4 ± 11.4, 17.9 ± 16.2) was not significantly different from those obtained with hrMRI (16.3 ± 8.4, 20.0 ± 10.3) (*p* = 0.932, *p* = 0.074) (Fig. 5).

#### Quantitative analysis: wall morphology features

Table 12 shows the ICC of wall morphological index measurements of the SBB *versus* hrMRI, demonstrating a very good reproducibility of the outer wall area, lumen area, wall area, remodeling index, plaque burden, maximum thickness, minimum thickness, and eccentricity relative to hrMRI, with good-to-excellent values for all plaque morphology-related parameters (ICC 0.85–0.94).

**Table 12:** Intraclass correlation coefficient (ICC) of remodeling index, plaque burden, maximal wall thickness, and eccentricity

	ICC	95% confidence interval	*p*-value
Remodeling index	0.94	0.91–0.97	< 0.001
Plaque burden	0.86	0.69–0.93	< 0.001
Maximal wall thickness	0.88	0.80–0.93	< 0.001
Eccentricity	0.85	0.76–0.91	< 0.001

## Discussion

A previous study [15] showed that posterior circulation stroke is more prevalent in the Asian population, which applies to our clinical cohort. Posterior circulation plaques accommodated a higher plaque burden before the formation of stenosis [16]. Perivascular CSF around posterior circulation provides high contrast in the background for the measurement of intracranial arterial boundary. Based on these considerations, we focused our current experimental study on the assessment of three segments (left vertebral V4 segment, right vertebral V4 segment, and basilar artery).

Spectral CT technology, especially when using a dual-layer detector, can provide a wealth of information without any change in the acquisition workflow and patient management; the derived data can be used to generate both true conventional and spectral results, as well as customized material decomposition results. We utilized the novel SBB approach to improve the diagnostic certainty in the depiction of the intracranial arterial wall, conspicuity, plaque detectability, and accuracy of plaque morphology measurements.

The phantom study showed that a tube wall with a 1-mm thickness could not be detected on conventional images and became detectable in the SBB reconstructions (see Fig. 1b, c). This is due to the blooming artifact caused by the iodinated contrast concealing the intima side of the tube in a 1-mm tube wall in conventional reconstructions. Moreover, while wall thicknesses > 2 mm could be detected in both conventional and SBB images, the quantitative measurement of wall thickness was significantly overestimated in the conventional images (MAE 8%) compared to those measured using the SBB reconstructions (MAE 3%). Also, the tube inner diameter was significantly underestimated in conventional images (MAE 19%) compared to SBB (MAE 2%). These results can be attributed to the fact that blooming artifact brings the false appearance of “intima ingression” visually in greater than > 1-mm tube walls. Our phantom results indicate that the material decomposition method has the potential to increase the accuracy of lumen stenosis measurements by up to eightfold in small vessels. The ratio between lumen CNR and the wall CNR of SBB images was significantly higher than in the conventional CTA images, with the same trend in the clinical study (Table 5 and Fig. 2).

CT blooming artifact refers to distortion or artifact in CT images that occurs due to the high density of certain materials, such as metal or iodinated contrast agents [17]. These materials can cause the CT scanner to overestimate the amount of radiation absorbed by the surrounding tissues, resulting in inaccuracies [18]. Material decomposition may help differentiate iodinated contrast agents from the surrounding intima of arterial vessels and reduce blooming artifacts. Iodinated contrast agents are often used in CTA to better visualize the blood vessels. However, since these agents have very high attenuation, they can cause blooming artifacts that can obscure the surrounding vessel walls and lead to inaccurate diagnoses. Material decomposition can help differentiate iodinated contrast agents from the surrounding tissues by analyzing and calculating the attenuation values of each material present in the image and reducing partial volume effects that can contribute to the severity of these artifacts [17]. Also, spectral CT reconstruction can reduce image noise and increase contrast resolution and thus improve CNR as shown in prior work involving both phantom and clinical data [19]. It can help to improve image quality and detectability of relatively small structures, such as the intracranial vessel walls.

The clinical study part demonstrated that SBB images increase the diagnostic certainty and conspicuity of intracranial vessels over conventional CTA images with good to excellent inter- and intra-reader agreement. After training, it improved the performance of inexperienced readers (sensitivity, specificity, and accuracy for all plaque sizes), almost to the level of experienced radiologists. However, the result remained similar before and after training for conventional images. We used segment-based weighting to quantify plaque detectability. This approach enabled us to provide insights into the segment level, since otherwise segments with more plaques would have been weighted more than normal segments. With this normalization, the accuracy measures such as NPV and PPV better reflect the severity of disease at a segment level. SBB images overcome conventional CTA limitations by increasing the CNR_wall/lumen_ to a value of (normal/abnormal artery, 78.3 ± 50.4/79.3 ± 96.7), higher than what was measured in hrMRI (normal/abnormal artery, 18.9 ± 8.4/24.1 ± 14.1) (see Fig. 5), thus enhancing the accuracy for plaque detectability from typically < 0.5 in conventional images to > 0.8 in SBB (see Table 11), similar to hrMRI. The phenomenon of high CNR_wall/lumen_ was similar to what was observed in the phantom study.

The difference in CNR_wall/periarterial_ CSF between hrMRI and SBB was not significant, but the *p*-value (0.074) was borderline may be due to the small sample size and thus requires further investigation. Currently, it is at least not inferior to hrMRI. Clinically speaking, while hrMRI can depict well vessel wall to surrounding tissue, the SBB in the current study can provide similar clarity of vessel wall to periarterial CSF. Measurements of the morphological characteristics (remodeling index, plaque burden, eccentricity) of SBB displayed no significant difference compared to hrMRI (ICC 0.94, 0.86, and 0.85, respectively) (Table 12). Thus, spectral CT holds promise for the early detection of morphological changes in intracranial arteries, providing early warning to clinics to help reduce disability and mortality, without the additional burden of radiation dose compared to conventional CTA. It has the potential to reveal the culprit plaque causing stroke in early CTA images without the presence of obvious stenosis. We used cross-sectional cuts perpendicular to the central line of the lumen in the curved multiplanar reconstruction mode to evaluate the vessel wall to minimize any false appearances of eccentric plaques which could occur with the use of oblique planes.

The phantom experiments showed that the use of SBB could significantly improve the accuracy of inner tube diameter measurement compared with conventional CTA (MAE from 19% down to 2%). The use of this new spectral visualization has the potential to increase the precision of lumen stenosis measurement, influencing the clinical intervention guidelines for cardiovascular disease. However, the spectral algorithm still has room for further adjustment, *e.g.*, for aspects such as the contrast between the vessel wall and surrounding fatty tissue, etc. Additional large-scale clinical trials are needed before becoming mainstream in clinical practice.

One important note is that 42 out of 103 cases were excluded due to motion artifacts in hrMRI. CT only takes a few seconds to acquire, which has a significant advantage over MRI. This inherent benefit of CT makes it more suitable and practical for plaque morphology assessment in stroke patients.

The novel SBB reconstruction also has the potential to be applicable in other iodine-enhanced cardiac structures such as the coronary arteries, carotids, and cardiac chambers. With its high CNR helping in determining the inner and outer boundary of the artery/tube, it could facilitate the use of artificial intelligence-based approaches for the automatic segmentation of vessels and chambers, further paving the way for calculations and simulations of non-invasive blood flow (such as the CT-based calculation of the fractional flow reserve) for use in clinical routine.

Our study had several limitations. First, the sample size was relatively small. It is necessary to prove its effect in daily practice with larger patient cohorts. Second, the *in vivo* composition of the plaque may not perfectly resemble the *ex vivo* plaque composition used in the phantom model: we used a light modeling resin that is routinely used in medical 3D printing practice. Third, this study was carried out on a dual-layer spectral CT platform. Further studies need to be undertaken using source-based dual-energy platforms, such as kVp switching and dual-source CT. Fourth, we did not include calcified or mixed plaques because it is hard to adjust the level of calcium suppression and the suppression may lead to the misclassification of superficial calcified nodules as a continuation of the lumen or, even worse, as an arterial ulceration, although we observed that SBB could suppress both iodine and calcium in the vessel wall. This aspect needs further exploration. Lastly, this study was carried out on posterior circulation, and further studies on anterior circulation need to be explored for further development of the technique.

In conclusion, we demonstrated that the use of novel SBB CT reconstructions can decrease blooming artifacts and image noise and increase image contrast resolution along with the ability to detect and characterize intracranial vessel atherosclerotic disease with an accuracy similar to hrMRI. This capability has the potential to enable early and timely detection and diagnosis of possible culprit plaques that cause stroke, thereby increasing the awareness of early prevention and early intervention of atherosclerosis, especially those impacting critical organs such as the brain.

### Supplementary Information

Additional file 1: Fig. S1. Schematic view of the decomposition approach. Each point in the scatter plot represents a voxel in the acquired anatomy (the two axes reflect two orthogonal measurements that come out of the spectral-CT data). Each voxel is being projected (in a constant direction) to the air-water line, to create the SBB image.

## Data Availability

All data generated or analyzed during this study are included in this published article and its supplementary information files.

## Abbreviations

3D: Three-dimensional
CNR: Contrast-to-noise ratio
CSF: Cerebrospinal fluid
CT: Computed tomography
CTA: CT angiography
CV: Coefficient of variance
hrMRI: High-resolution magnetic resonance imaging
HU: Hounsfield unit
ICC: Intraclass correlation coefficient
IQR: Interquartile range
MAE: Mean absolute error
ROI: Region of interest
SBB: Spectral black-blood
SD: Standard deviation
