# Supplementary material for: Detectability of intracranial vessel wall atherosclerosis using black-blood spectral CT: a phantom and clinical study
Source: Eur Radiol Exp. 2024 Jul 3;8:78. doi: 10.1186/s41747-024-00473-x (PMC11219652; doi:10.1186/s41747-024-00473-x)
Supplement: Supplementary file 1 — Additional file 1: Fig. S1. Schematic view of the decomposition approach. Each point in the scatter plot represents a voxel in the acquired anatomy (the two axes reflect two orthogonal measurements that come out of the spectral-CT data). Each voxel is being projected (in a constant direction) to the air-water line, to create the SBB image. [file 41747_2024_473_MOESM1_ESM.pdf]

Detectability of intracranial vessel wall atherosclerosis using black-blood spectral CT: a phantom and clinical study

ELECTRONIC SUPPLEMENTARY MATERIAL

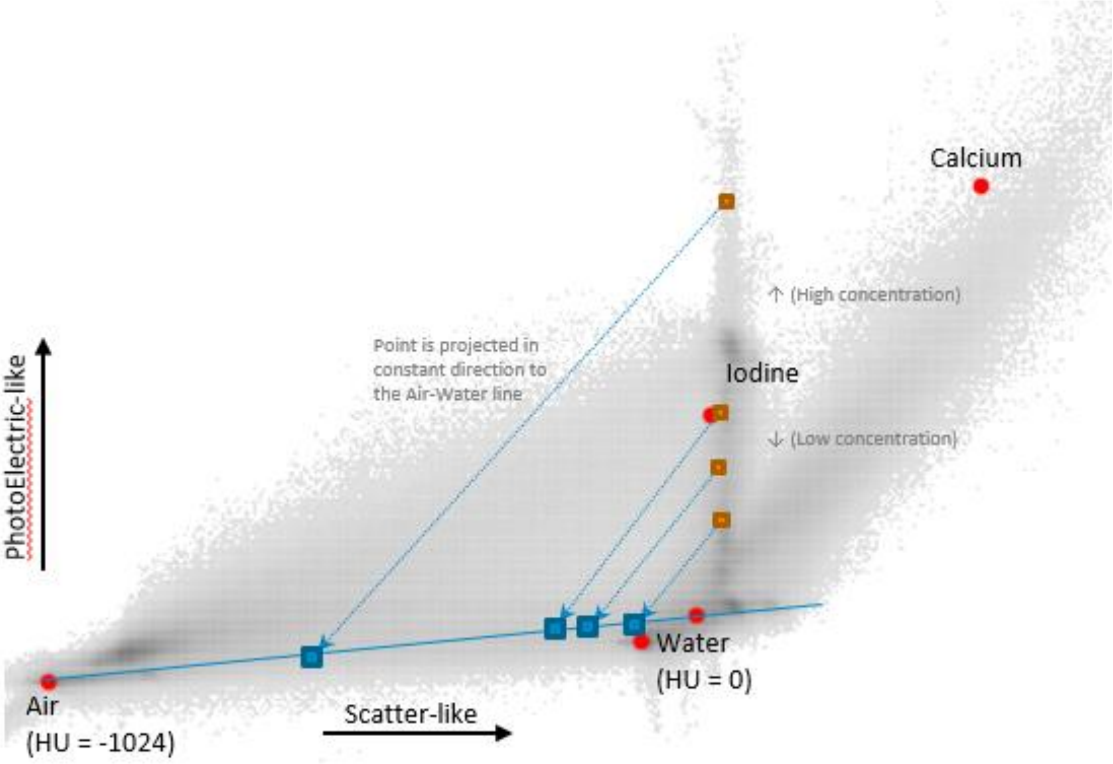

Figure. S1 Schematic view of the decomposition approach. Each point in the scatter plot represents a voxel in the acquired anatomy (the two axes reflect two orthogonal measurements that come out of the spectral-CT data); Each voxel is being projected (in a constant direction) to the air-water line, to create the SBB image.
